# Chitinase-Like (*CTL*) and Cellulose Synthase (*CESA*) Gene Expression in Gelatinous-Type Cellulosic Walls of Flax (*Linum usitatissimum* L.) Bast Fibers

**DOI:** 10.1371/journal.pone.0097949

**Published:** 2014-06-11

**Authors:** Natalia Mokshina, Tatyana Gorshkova, Michael K. Deyholos

**Affiliations:** 1 Kazan Institute of Biochemistry and Biophysics, Russian Academy of Sciences, Kazan, Russia; 2 Department of Biological Sciences, University of Alberta, Edmonton, Alberta, Canada; Iowa State University, United States of America

## Abstract

Plant chitinases (EC 3.2.1.14) and chitinase-like (CTL) proteins have diverse functions including cell wall biosynthesis and disease resistance. We analyzed the expression of 34 chitinase and chitinase-like genes of flax (collectively referred to as *LusCTLs*), belonging to glycoside hydrolase family 19 (GH19). Analysis of the transcript expression patterns of *LusCTLs* in the stem and other tissues identified three transcripts (*LusCTL19, LusCTL20, LusCTL21*) that were highly enriched in developing bast fibers, which form cellulose-rich gelatinous-type cell walls. The same three genes had low relative expression in tissues with primary cell walls and in xylem, which forms a xylan type of secondary cell wall. Phylogenetic analysis of the LusCTLs identified a flax-specific sub-group that was not represented in any of other genomes queried. To provide further context for the gene expression analysis, we also conducted phylogenetic and expression analysis of the cellulose synthase (CESA) family genes of flax, and found that expression of secondary wall-type *LusCESAs* (*LusCESA4, LusCESA7* and *LusCESA8*) was correlated with the expression of two *LusCTLs* (*LusCTL1, LusCTL2*) that were the most highly enriched in xylem. The expression of *LusCTL19, LusCTL20,* and *LusCTL21* was not correlated with that of any CESA subgroup. These results defined a distinct type of CTLs that may have novel functions specific to the development of the gelatinous (G-type) cellulosic walls.

## Introduction

Flax (*Linum usitatissimum* L.) phloem fibers are a valuable industrial feedstock and are also a convenient model system for studying secondary cell wall formation. The mechanical properties of bast fibers are largely dependent on the composition of their secondary walls. Bast fibers have gelatinous-type walls, which are rich in cellulose (up to 90%) and lack detectable xylan and lignin. Gelatinous fibers are widespread in various land plant taxa, but have been studied primarily in angiosperms. Depending on the species, either phloem or xylem (of either primary or secondary origin) can produce gelatinous fibers in various organs including stems, roots, tendrils, vines, and peduncles [1], [2]. The mechanisms of gelatinous cell wall development in these fibers remain largely unclear. However, some genes implicated in gelatinous cell wall development have been identified. The list includes fasciclin-like arabinogalactan proteins (FLAs) [3]–[6], β-galactosidases [7], [8], and lipid transfer proteins [6]. A role for β-galactosidases in G-type wall development has been demonstrated functionally [8].

Transcripts of genes encoding chitinase-like proteins are reportedly enriched in fibers, particularly during the cell wall thickening stage of flax phloem cellulosic fiber development [6]. Expression of CTLs during primary or secondary cell wall deposition has also been reported in species other than flax [9], [10]. Plant chitinase-like proteins have been identified in a wide range of organelles and tissues, including the apoplast and vacuole [11].

Chitinase-like proteins belong to a large gene family that includes genuine chitinases (i.e. proteins with proven chitinase activity) and other homologous proteins, which may not have chitinase activity [12]–[15]. Here, we refer to both genuine chitinases and their homologs collectively as chitinase-like proteins (CTLs).

Chitinases catalyze cleavage of β-1,4-glycoside bonds of chitin and are organized in five classes (Classes I–V), which can be distinguished on the basis of sequence similarity [11], [16], [17]. Classes I, II, and IV belong to glycoside hydrolase family 19 (GH19), found primarily in plants, whereas Classes III and V belong to glycoside hydrolase family 18 (GH18) present in various types of organisms [18]–[20]. The Class I chitinases are found in both monocots and dicots, while classes II and IV are found mainly in dicots [21]. Class I and IV chitinases contain a highly-conserved cysteine-rich domain – the chitin binding domain (CBD) – at the N- terminal region [21], [22], but there are two characteristic deletions in the main catalytic domain in Class IV chitinases [21]. Because chitin is the major component of fungal cell walls, chitinases are classic pathogenesis-related proteins involved in non-host-specific defense [23], [24].

Plants also contain chitinase-like proteins that are not induced by pathogens or stresses. In many cases, these chitinase-like proteins have been shown to lack detectable chitinase activity. Chitinase-like proteins may play an important role during normal plant growth and development [13], [15], [25]. For example, *AtCTL1* is constitutively expressed in many organs of *Arabidopsis*. Mutations of *AtCTL1* lead to ectopic deposition of lignin in the secondary cell wall, reduction of root and hypocotyl lengths, and increased numbers of root hairs [15]. It was suggested that this gene could be involved in root expansion, cellulose biosynthesis, and responses to several environmental stimuli [13], [26], [27]. In particular, co-expression of some CTLs with secondary cell wall cellulose synthases (CESAs) was reported [28]. It has been suggested that these chitinase-like proteins could take part in cellulose biosynthesis and play a key role in establishing interactions between cellulose microfibrils and hemicelluloses [14].

The xylan-type secondary wall is the most common secondary wall in land plants and is characteristically rich in cellulose, xylan, and lignin [2]. Compared to typical xylan-type secondary walls, gelatinous layers are enriched in cellulose, have a higher degree of cellulose crystallinity, larger crystallites, and a distinctive set of matrix polysaccharides (see [2] and references therein). Presumably, cellulose synthase genes have a significant role in gelatinous cell wall formation, but the expression patterns of the complete flax *CESA* family has not been described to date. It is known that at least three isoforms of CESAs comprise the cellulose synthase rosette: CESA1, CESA3, and CESA6 are required for cellulose biosynthesis in primary cell walls [29], whereas CESA4, CESA7, and CESA8 are required for cellulose biosynthesis during secondary wall deposition [30].

Flax is a useful model for comparative studies of cell wall development: different parts of the flax stem form a primary cell wall, xylan type secondary cell wall, or gelatinous cell wall; these stem parts may be separated and analyzed by diverse approaches, including functional genomics. Furthermore, the publication of a flax whole genome assembly [31] facilitates a thorough study of key gene families.

In the present study, we measured expression of all predicted *LusCTL* genes of the GH19 family in various tissues including those that produce gelatinous-type and xylan-type cell walls. We also described the *LusCESA* gene family and measured expression of its transcripts in comparison to *LusCTLs*. Phylogenetic analysis of *LusCTL* and *LusCESA* genes identified distinct groups of *LusCTL* genes that were expressed preferentially at specific stages of bast fiber gelatinous cell wall development.

## Materials and Methods

### Plant Growth

**Figure 1:**
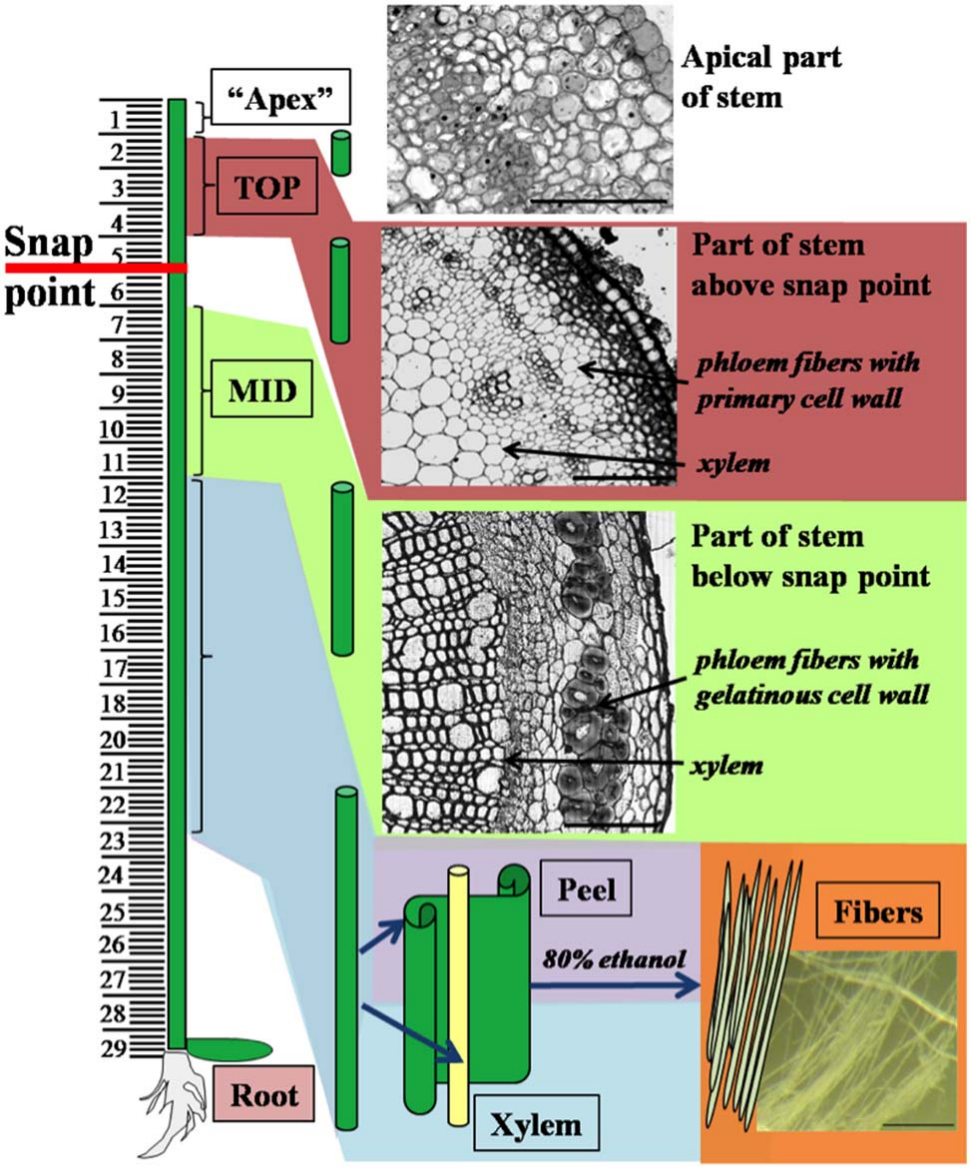
Scheme of sample collection. The segments of the flax stem (apical part “Apex”, top region TOP, middle region MID, Peel containing phloem fibers, Xylem and isolated Fibers) were taken for RNA extraction. A detailed description of each sample is presented in Table 1. Bar: 50 µm for “Apex”, 100 µm for TOP and MID, 1 mm for Fibers.

Flax (*Linum usitatissimum* L.) var. Mogilevsky plants were grown in pots in a growth chamber at 22°C, with a light intensity of approximately 200 µE on a 16 h light/8 h dark cycle. Plants were harvested at the period of rapid growth (4 weeks after sowing). Plant material was sampled with respect to the location of the snap point, which is a mechanically defined stem position in which fibers undergo transition from elongation to secondary cell wall formation [32]. The following seven samples were collected (Table 1, Figure 1): 1. “Apex” – the apical part of stem (1 cm of length). 2. “TOP” – the following “apex” segment of stem above the snap point with phloem fibers in the process of elongation. 3. “MID” – the stem segment (5 cm of length) below the snap point which contained fibers at early stages of secondary cell wall thickening. 10 cm of the stem downwards from “MID” was divided into Peel (4), which contained epidermis, parenchyma cells, phloem fiber bundles and sieve elements and Xylem (5), which contained parenchyma cells, xylem vessels and xylem fibers. 6. “Fibers” – i.e. isolated phloem fibers were obtained by washing Peels in 80% ethanol in a mortar several times and gently pressing the fiber-bearing tissues with a pestle to release the fibers. 7. Roots. The number of biological replicates was three, with five plants in each replicate.

**Table 1:** Description of plant samples.

Sample	Basic tissues	The main difference from other tissues
“Apex”	epidermis, meristem cells, parenchyma cells, procambium, sieve elements, companion cells, fast growing phloem fibers with primary cell wall, xylem vessels with primary cell wall and secondary thickness appears	phloem fibers with primary cell wall
“TOP”	epidermis, parenchyma cells, sieve elements, companion cells, fast growing phloem fibers with primary cell wall, xylem fibers and vessels secondary thickness (xylan cell wall)	phloem fibers with primary cell wall
“MID”	epidermis, parenchyma cells, sieve elements, companion cells, cambium, phloem fibers with gelatinous cell wall, xylem fibers and vessels with xylan cell wall	phloem fibers at the beginning of gelatinous cell wall deposition
“Peel”	epidermis, parenchyma cells, sieve elements, companion cells, fibers with thick gelatinous cell wall	phloem fibers with thick gelatinous cell wall
“Xylem”	parenchyma cells, cambium, xylem fibers and vessels with xylan cell wall	xylem fibers and vessels with xylan type of cell wall
“Fiber”	isolated fibers with thick gelatinous cell wall	isolated phloem fibers with thick gelatinous cell wall
“Root”	epidermis, parenchyma cells, sieve elements, companion cells, xylem fibers and vessels with xylan cell wall	xylem fibers and vessels with xylan type of cell wall

### Sequence Alignment and Phylogenetic Analysis

Predicted amino acid and nucleotide sequences of CTLs (Pfam domain: PF00182) and CESAs (PF03552) were obtained from the Phytozome database v.9.0 (*Linum usitatissimum*, *Populus trichocarpa*, *Arabidopsis thaliana*). CESAs of poplar (PtiCESAs) were renamed according to Kumar et al. [33]. A list of various well-characterized CTLs from different plant species was obtained from previously published works [10], [21]. Sequences were aligned using MUSCLE with default parameters, and a phylogenetic tree was constructed using MEGA5 based on the Maximum Likelihood and Neighbor-Joining methods [34], bootstrapping 1000 replicates [35], model WAG+G or JTT+G. Signal peptides for protein sequences were predicted using SignalP (http://www.cbs.dtu.dk/services/SignalP/), molecular weights, isoelectric points of the proteins were analyzed by ProtParam (http://web.expasy.org/protparam/).

### Reverse Transcription Quantitative Real Time PCR

Total RNA from all plant samples was isolated using a Trizol-extraction method combined with an RNeasy Plant Mini Kit (Qiagen) according to the manufacturer’s instructions. RNA quality was evaluated by electrophoresis using a BioAnalyzer (Agilent), and no degradation of RNA was evident. Residual DNA was eliminated by treatment with DNAse I using the DNA-free kit (Ambion). Gene specific primers for CTL and CesA genes were designed using Universal ProbeLibrary Assay Design Center (http://www.roche-applied-science.com/shop/CategoryDisplay?catalogId=10001&tab=&identifier=Universal+Probe+Library) (File S1). One microgram of total RNA was reverse-transcribed using RevertAid H Minus First Strand cDNA Synthesis Kit (Thermo Scientific). The cDNAs were diluted 1∶32 with nuclease free water. Real-time PCR was performed in a 7900 HT Fast real-time PCR system (Applied Biosystems, USA). Each 10 µL real-time PCR cocktail contained 2.5 µL of 0.4 µM concentrations of both forward and reverse gene-specific primers, and 2.5 µL of cDNA, 5 µL of 2×Dynamite qPCR mastermix (Molecular Biology Service Unit - University of Alberta) which included SYBR green (Molecular Probes) and Platinum Taq (Invitrogen). The thermal cycling conditions were 95°C for 5 minutes, 40 cycles of 95°C for 15 seconds, and 60°C for 1 minute. A 60–95°C melting curve was performed to confirm the specificity of the products. Threshold cycles (CT) were determined using 7900 Fast Software. C_T_ values were normalized using eukaryotic translation initiation factors 1A, 5A (*LusETIF1, LusETIF5A*) and glyceraldehyde 3-phosphate dehydrogenase (*LusGAPDH*) gene from flax (File S1) [36]. From each of three biologically independent cDNA samples, two independent technical replications were performed and averaged for further calculations. ΔΔCT values were generated using the apex sample as a reference. Relative transcript abundance calculations were performed using comparative C_T_ (ΔC_T_) method as previously described [37] for flax tissues (TOP/Apex, MID/Apex, Peel/Apex, Xylem/Apex, Fiber/Apex, Root/Apex). Heat maps of expression levels of some genes were then created with MeV v4.8 (Multi Experiment Viewer, http://www.tm4.org/mev.) using the means of ΔC_T._

## Results

### *LusCTL* Phylogenetic Characterization

We searched within the flax genome assembly (version 1.0) for predicted genes with homology to Pfam domain PF00182, which is characteristic of chitinases of the glycosyl hydrolase family 19 (GH 19) family [31], [38]. This search identified 37 predicted chitinase or chitinase-like genes (referred to here collectively as *LusCTL*s) (Table 2). However, only three of the predicted proteins (*LusCTL8, LusCTL10*, and *LusCTL15*) contained a conserved chitin-binding domain (CBD), suggesting that not all of the LusCTLs use chitinase as a substrate. The mean predicted protein size of the 37 *LusCTLs* was 246.5 aa (or 27 kDa), and the majority (30/37) were predicted to be secreted (Table 2).

**Table 2:** Predicted chitinase-like sequences in *L. usitatissimum.*

Locus I.D.	Label	Length, aa	MW, kDa	pI	Domains*	SP**	Group
Lus10016872	LusCTL1	209	22.9	6.2	−	−	A
Lus10037737	LusCTL2	330	35.9	6.7	−	+	A
Lus10037428	LusCTL3	327	36.2	7.0	−	+	A
Lus10037430	LusCTL4	325	36.0	6.7	−	+	A
Lus10041278	LusCTL5	325	35.9	6.7	−	+	A
Lus10041282	LusCTL6	118	13.2	5.2	−	−	A
Lus10041829	LusCTL7	131	14.5	7.7	−	−	B
Lus10041830	LusCTL8	320	34.4	6.9	CBD	+	B
Lus10028378	LusCTL9	131	13.8	6.0	−	−	B
Lus10028377	LusCTL10	328	35.1	7.4	CBD	+	B
Lus10041831	LusCTL11	125	13.6	9.0	−	−	B
Lus10000193	LusCTL12	125	13.5	8.6	−	−	B
Lus10038026	LusCTL13	274	30.2	8.8	−	+	B
Lus10009968	LusCTL14	274	30.2	8.9	−	+	B
Lus10000453	LusCTL15	264	28.7	4.4	CBD	+	C
Lus10003230	LusCTL16	235	26.4	8.4	−	+	C
Lus10024367	LusCTL17	223	24.6	8.5	−	+	C
Lus10010863	LusCTL18	232	25.6	8.6	−	+	C
Lus10010864	LusCTL19	230	25.8	8.6	−	+	C
Lus10010866	LusCTL20	224	24.8	5.1	−	+	C
Lus10024366	LusCTL21	225	24.8	5.1	−	+	C
Lus10035618	LusCTL22	232	25.5	8.9	−	+	C
Lus10035620	LusCTL23	389	43.4	9.0	−	+	C
Lus10003231	LusCTL24	226	24.8	9.2	−	+	C
Lus10035621	LusCTL25	414	44.7	7.1	−	+	C
Lus10024369	LusCTL26	226	25.1	8.7	−	+	C
Lus10035624	LusCTL27	318	35.7	5.7	−	+	C
Lus10003227	LusCTL28	317	35.6	5.7	−	+	C
Lus10000217	LusCTL29	193	21.6	4.4	−	+	C
Lus10035625	LusCTL30	304	33.7	6.1	−	+	C
Lus10003226	LusCTL31	326	36.2	4.9	−	+	C
Lus10024368	LusCTL32	229	25.0	9.9	−	+	C
Lus10010861	LusCTL33	229	24.9	9.9	−	+	C
Lus10010862	LusCTL34	229	25.0	9.9	−	+	C
Lus10003587	LusCTL35	223	24.1	9.5	−	+	C
Lus10032794	LusCTL36	223	24.1	9.5	−	+	C
Lus10010860	LusCTL37	69	8.1	9.7	−	−	C

*presence of predicted domains in addition to Glyco_hydro_19 domain.

**predicted secreted protein (according to TargetP).

The labels assigned to the 37 predicted *LusCTLs* are shown in Table 2. *LusCTL1* and *LusCTL2* were so named because they encoded proteins that were most similar to *CTL1* and *CTL2*, respectively, which have been characterized in other species (e.g. *A. thaliana* [14] and *G. hirsutum* [10]) (Table 2). The gene *LusCTL37* was predicted to encode only a protein of 69 aa, which is much shorter than the rest of the LusCTLs (Table 2), and so it was not used in further analyses.

**Figure 2:**
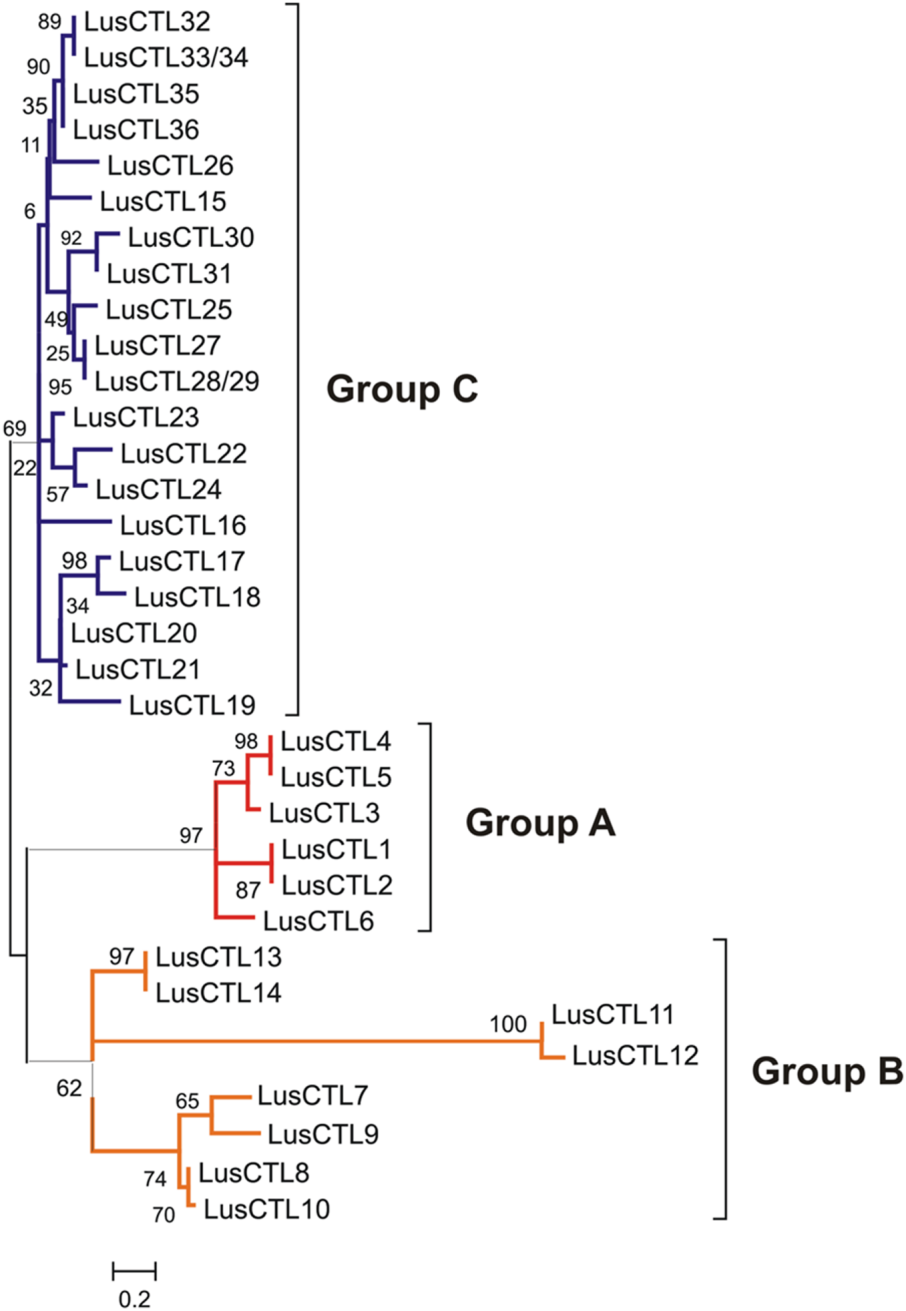
Phylogenetic relationships among *LusCTL*s. All predicted *LusCTL*s amino acid sequences were aligned using their deduced full-length peptide sequences in the MEGA platform (MUSCLE), Maximum Likelihood Method, JTT+G model, bootstrap 1000.

**Figure 3:**
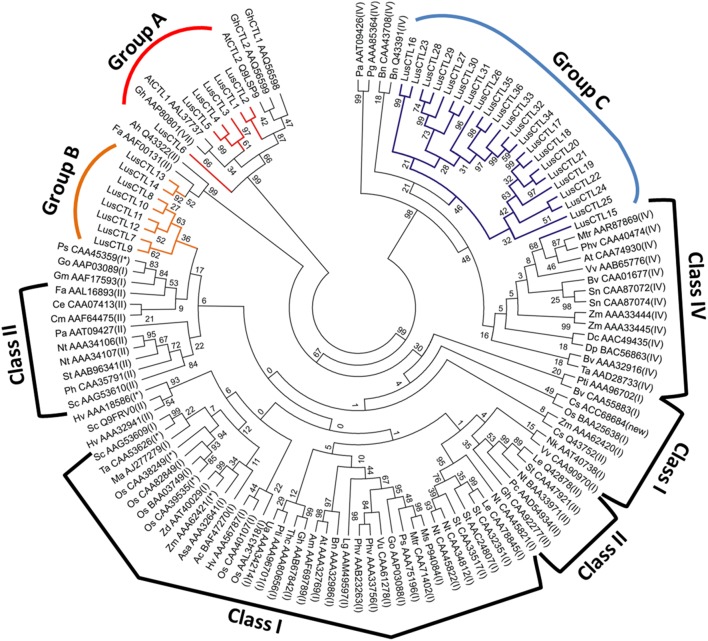
Phylogenetic relationship among *LusCTL*s and selected CTLs of 43 different plant species. All the predicted CTLs sequences were aligned using their deduced full-length peptide sequences in MEGA (MUSCLE). Maximum Likelihood Method, WAG+G model, bootstrap 1000. Classes I–IV were labeled according to Hamel et al. [21] and Zhang et al. [10] and Groups A-C are as shown in Figure 2.

The LusCTLs and their inferred phylogenetic relationships are shown in Figure 2. Based on this dendrogram, the predicted LusCTLs were divided into three groups: Group A included *LusCTL1–6*, Group B included *LusCTL7–14*, and Group C included *LusCTL15–36* (Figure 2, Table 2). The flax-specific tree shown in Figure 2 was expanded by the addition of representative GH19 CTLs from other species (Figure 3). In this multispecies tree, LusCTLs of Group A, which includes *LusCTL1* and *LusCTL2*, were part of the same clade as the well-characterized *AtCTL2* of *A. thaliana* and *GhCTL1*, *GhCTL2*, *GhCTLVII* of *G. hirsutum,* The Group B *LusCTLs* (*LusCTL7–14*) were in the same clade as the previously defined Classes I, II, III, GH19 chitinases [10], [21]. Most of group B was in the same sub-clade as Class II, although none of the previously defined Classes I–III were monophyletic in our analysis. Finally, our Group C *LusCTLs* (*LusCTL15–36*) formed a monophyletic clade with representatives of the previously defined Class IV GH19 chitinases.

### *LusCTL* Transcript Expression

**Figure 4:**
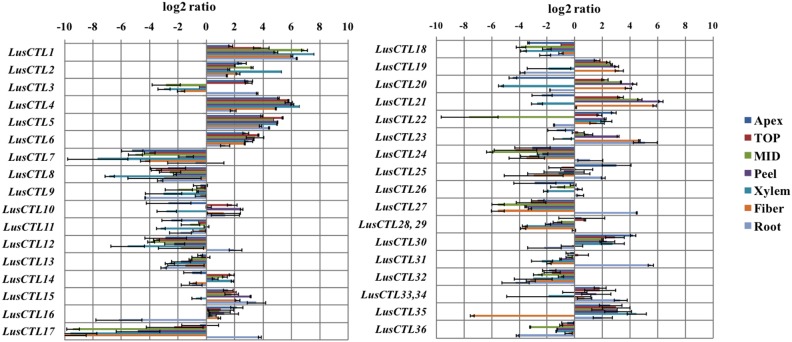
Relative differential expression of *LusCTL* genes in different tissues of the flax stem. ΔΔC_T_ values for each of CTLs were generated using an average ΔC_T_ for all CTLs as a reference. Error bars show the standard error of the mean.

Quantitative real-time reverse-transcription PCR (qRT-PCR) was performed to study *LusCTL* expression patterns of *L. usitatissimum* genes of chitinase-like proteins in various tissues and stages of development (Table 1). These tissues and their names as used here are equivalent to the names used in previous studies [6], [32], [39], [40]. Only 34 sets of primers were used in this assay, because members of each of two pairs of *LuCTLs* could not be distinguished by unique primers: *LusCTL28* and *LusCTL29* (95.6% aa and 96.3% nt identity), and *LusCTL33* and *LusCTL34* (99.6% aa and 98.8% nt identity). Thus a common set of primers was used for each of these pairs. We observed that transcripts of *LusCTL1* showed enriched levels of expression (compared to the apical part of stem) in tissues in which cell walls were undergoing thickening (Figure 4) in xylem and in phloem fibers. Transcripts for this gene were enriched 57-fold in xylem, 28-fold in the MID region, and 20-fold in fiber. Another predicted CTL, *LusCTL2*, showed a similar pattern of enrichment in secondary-wall bearing tissues (8.3, 4.5 and 1.4-fold higher in xylem, MID and fiber, respectively, compared to the apex), although the magnitude of its enrichment was not as strong as *LusCTL1*. These two *LusCTLs* had high sequence similarity to each other (91.9% amino acid identity) and had similar patterns of expression as compared to each other in the various flax tissues.

A subset of *LusCTL* genes (*LusCTL10*, *LusCTL11, LusCTL19, LusCTL20, LusCTL21, LusCTL23, LusCTL24, LusCTL26*) had high relative expression in tissues that contained phloem fibers (MID, peel, fiber) but low relative expression in xylem (Figure 4). Three of these genes (*LusCTL19*, *LusCTL20, LusCTL21*) were enriched >40-fold in fibers compared to the apical part of stem. These three genes had high similarity to one another (76% identity between *LusCTL19* and *LusCTL20* as well as between *LusCTL19* and *LusCTL21*; 91% identity between *LusCTL20* and *LusCTL21*) (Figure 2).

### *LusCESA* Phylogenetic and Expression Characterization

**Figure 5:**
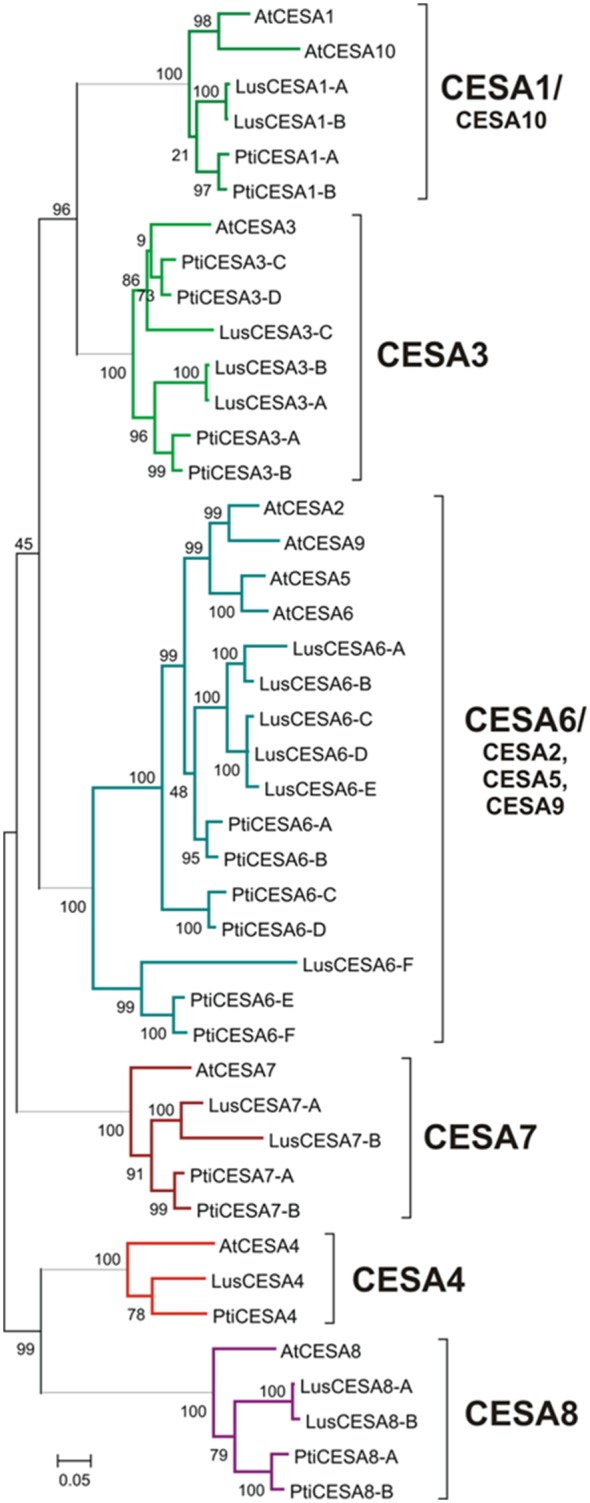
Phylogenetic relationship among *LusCESAs*, *AtCESAs*, *PtiCESA*. All CESAs sequences were aligned using their deduced full-length peptide sequences on MEGA platform (MUSCLE). Maximum Likelihood Method, JTT+G model, bootstrap 1000.

To provide context for the expression patterns of *LusCTLs,* and to test whether the expression pattern of cellulose synthase (*LusCESA*) genes differed between gelatinous fibers and cells with a xylan type of secondary cell wall, expression of *LusCESAs* in different flax tissues was analyzed. We identified 14 predicted *LusCESAs* in the flax whole genome assembly by searching predicted proteins for the conserved cellulose synthase domain (Pfam PF03552). No putative *LusCESA7* genes were found in the original published genome published (v1.0, [31]). However, though BLAST alignment of the CDS of *Arabidopsis* and poplar CESA7 sequences, two scaffolds (scaffold_57 and scaffold_464) of the flax genome assembly were identified as encoding CESA7 homologs, and these were annotated using the Augustus server (http://bioinf.uni-greifswald.de/augustus/). Thus, all 16 predicted *LusCESAs* were aligned with well-characterized *AtCESAs* from *A. thaliana* and *PtiCESAs* from *P. trichocarpa* (Figure 5). This alignment was used to construct a phylogenetic tree and annotate the *LusCESAs*, which were named according to the established *A. thaliana* [41] and *P. trichocarpa* nomenclature systems (Table 3, [33]). The number of *LusCESAs* and *PtiCESAs* isoforms identified for each of the eight major types of CESAs was similar except in the case of CESA3, where one more gene was identified in *P. trichocarpa* than in *L. usitatissimum* (Figure 5, Table 3). The *LusCESA* appeared to be typical of other genes in this family in that they were large integral membrane proteins with eight predicted transmembrane domains, a hydrophilic domain that faces the cytosol, and a zinc finger domain at the N-terminus of proteins with the characteristic amino acid motif “CxxC” (specific for CESAs only [42]).

**Table 3:** Predicted cellulose synthase sequences in *Linum usitatissimum*.

Locus I.D.	Code	Pti homolog	At homolog	Length, aa	MW, kDa	pI
Lus10018902	LusCESA1-A	PtiCESA1-A,	AtCESA1	1079	121.7	6.3
Lus10028597	LusCESA1-B	PtiCESA1-B	(AtCESA10)	1079	121.7	6.3
Lus10039607	LusCESA3-A	PtiCESA3-A,	AtCESA3	1069	120.2	8.0
Lus10007538	LusCESA3-B	PtiCES3-B,		1092	122.3	7.5
Lus10012198	LusCESA3-C	PtiCES3A–C, PtiCES3A–D		1094	122.6	7.0
Lus10008225	LusCESA4	PtiCESA4	AtCESA4	987	111.3	6.9
Lus10006161	LusCESA6-A	PtiCESA6-A,	AtCESA6	1074	121.4	6.8
Lus10041063	LusCESA6-B	PtiCESA6-B,	(AtCESA2,	1096	123.7	6.8
Lus10003526	LusCESA6-B	PtiCESA6-C,	AtCESA5,	1097	123.7	6.8
Lus10002939	LusCESA6-D	PtiCESA6-D,	At CESA9)	1097	123.5	7.1
Lus10002940	LusCESA6-E	PtiCESA6-E,		906	102.1	7.3
Lus10022449	LusCESA6-F	PtiCESA6-F		1035	116.9	6.1
scaffold_57	LusCESA7-A	PtiCESA7-A,	AtCESA7	978	111.2	6.8
scaffold_464	LusCESA7-B	PtiCESA7-B		959	108.3	8.6
Lus10007296	LusCESA8-A	PtiCESA8-A,	AtCESA8	988	111.4	6.4
Lus10029245	LusCESA8-B	PtiCESA8-B		988	111.3	6.7

**Figure 6:**
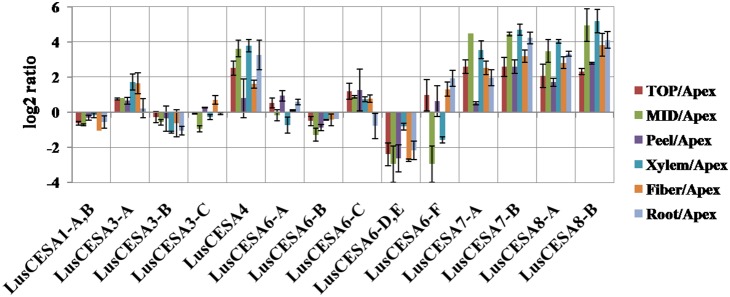
Relative expression of *LusCESAs* genes in different tissues of the flax stem. ΔΔC_T_ values were generated using the “Apex” sample as a reference. Error bars show the standard error of the mean.

Relative differential expression of *LusCESA* genes in different tissues of the flax stem was estimated (Figure 6). *LusCESA4, LusCESA8-A, LusCESA8-B, LusCESA7-A, LusCESA7-B* had high expression in tissue that produce secondary walls (TOP, MID, Xylem, Fiber, Root). Transcripts of these *LusCESA* isoforms were the most enriched in Xylem, which contained cells with xylan-type cell walls, and in roots, where secondary vascular tissue (xylem) was also well-developed. These secondary cell wall type *LusCESAs* had also high relative expression in cellulosic fibers, although it was not as strong as for xylem.

**Figure 7:**
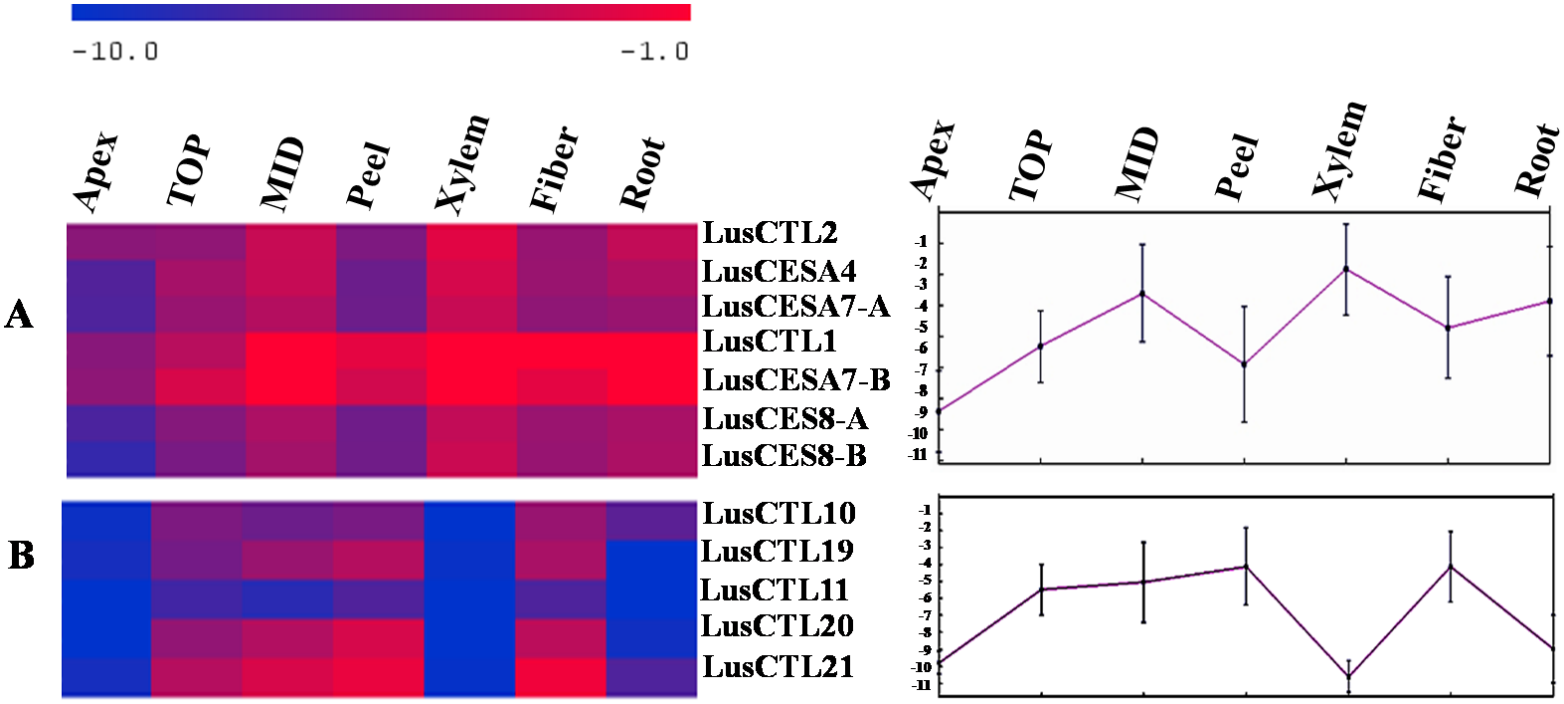
Expression of secondary wall LusCESAs and LusCTLs. A - Relative differential expression of secondary cell wall *LusCESAs 4, 7A, 7B, 8A, 8B* and *LusCTL1* and *LusCTL2* in different tissues of the flax stem and centroid graph (from the left) for the mean of ΔC_T_ of these 7 genes. B - Relative differential expression of fiber-enriched *LusCTLs* (*LusCTL10, 11, 19, 20, 21*) in different tissues of the flax stem and centroid graph (from the left) for the mean of ΔC_T_ of these 5 genes. The mean of ΔC_T_ of *LusCTLs* and *LusCESAs* were used to produce a heat map.

Changes in expression of the *LusCESA4, 7, 8* isoforms and “xylem-specific” *LusCTL1* and *LusCTL2* were well-correlated in different flax tissues (Figure 7A). This group of genes was highly expressed in tissues with secondary cell walls (MID, Xylem and Roots). In contrast, the “fiber-specific” *LusCTLs* had very different patterns of expression in the same tissues (Figure 7B): these had low level of expression in xylem, but high level of relative expression in tissues with gelatinous fibers (peel and fiber).

## Discussion

Certain fibers of many plant species form G-type cell walls, which are rich in crystalline cellulose [1]. Expression of CTLs has been previously reported to be enriched during development of G-type cell walls, along with specific FLAs [4], [6], [7], LTPs [6] and BGALs [6]–[8]. In this work, we analyzed expression of all *LusCTL* genes of GH 19 in different flax tissues and compared this expression with *LusCESAs* and to their inferred phylogenies.

In the flax genome, 16 predicted *LusCESAs* were identified (Table 3). Previously only partial sequences of some flax CESAs were published [43]. All 16 flax CESAs could be placed in discrete clades with *Arabidopsis* and *Populus* CESA homologs (Figure 5). We generally numbered *LusCESAs* in a way that reflects the association of each flax gene with its nearest relative in the *Arabidopsis* genome, as was done for CESAs of *Populus* [33]. Following this pattern, the *LusCESA6A–F* genes we named as a group, similar to *PtiCESA6A–F* and were not distinguished as *CESA2/9/5/6* as in *Arabidopsis* clade (Table 3) [44]. Most of the flax and *Populus CESA* genes are present as pairs of paralogs in their respective genomes, although there were three *LusCESA3* genes (*LusCESA3A–C*) for only two *Populus* genes and one Arabidopsis gene. *AtCESA1* and *AtCESA10* were represented by only one pair of genes (*LusCESA1A*, *B*) in flax.

It is well established that proteins encoded by different sets of three CESA genes (CESA1, 3, 6 and CESA4, 7, 8) are required for cellulose synthesis during primary and secondary wall formation, respectively [44]–[46]. The functional relationships of the various paralogs of *LusCESA*s (except *LusCESA4*, Table 3) are presently unclear. According to the data obtained here, secondary cell wall *LusCESA4, LusCESA7-A, B* and *LusCESA8-A, B* were highly expressed both in the xylem cells with lignified cell walls (i.e. xylan type) and in the phloem fibers with thick gelatinous cell wall. This suggests that phloem fibers and xylem may use similar, rather than specialized rosettes. This is consistent with observations from poplar showing only minor differences in expression of cellulose biosynthetic genes in tension wood as compared to normal wood [3]. The different properties of gelatinous and xylan type cell walls are therefore likely determined not by CESAs, but by other proteins associated with cellulose synthesis, which could include specific CTLs.

We observed two *LusCTLs* that were expressed more strongly in xylem tissue than in any other tissue surveyed (Figure 3, *LusCTL1, LusCTL2*). The co-expression of certain isoforms of *LusCTL1, LusCTL2* and the secondary wall *LusCESAs* (*CESA4, 7, 8*) suggested a role for these LusCTLs in secondary cell wall development (Figure 7). As noted above, *LusCTL1* and *2* are highly similar to *AtCTL2* of *A. thaliana* and *GhCTL1*, *GhCTL2*, of *G. hirsutum*. The role of *CTL2*, and its close homolog *CTL1*, in cell wall biosynthesis is especially intriguing since associations between CTLs and primary or secondary cell wall synthesis have been reported in different plant species [10]. *CTL2* is strongly upregulated during secondary wall formation in interfascicular fibers in *A. thaliana*. Reduction in crystalline cellulose content in *ctl1 ctl2* mutants was demonstrated, leading to the to the suggestion that *AtCTLs* are involved in cellulose assembly. Furthermore, in *P. trichocarpa*, expression of chitinase genes related to *AtCTL1*, *AtCTL2*, and *GhCTLVII* are highly correlated with secondary wall formation of xylem [47]. It has therefore been proposed that CTL1 and CTL2 work in conjunction with primary- and secondary-cell wall CESAs, respectively [14]. One of the hypotheses for CTL1/2 function is regulation of cellulose assembly and of interaction with hemicelluloses via binding to emerging cellulose microfibrils [14]. However, the mechanism of CTL action in cell wall biosynthesis as well as substrates of catalytic activity (if any) remains unknown. It was suggested that the likely substrates of plant chitinases may be arabinogalactan proteins, chitooligosaccharides and other GlcNAc-containing glycoproteins or glycolipids [13], [15], [48], [49] and the mechanism by which CTLs act is more likely to involve binding of chitin oligosaccharides than catalysis. Also, it is assumed that chitinases may participate in the generation of such signal molecules that regulate the organogenesis process [50].

Although relative expression of *LusCESA* (*4, 7, 8*) and *LusCTL1, LusCTL2* in xylem tissue was higher compared with phloem fibers, we cannot exclude involvement of these LusCTLs in phloem fiber cell wall development. At the same time, a distinct group of *LusCTLs* (*LusCTL19, LusCTL20, LusCTL21*) had very high enrichment in samples with phloem fibers (MID, peel, fiber) with a low level of expression in xylem. According to the phylogenetic tree, these LusCTLs (group C) were most similar to the previously defined Class IV chitinases (Figure 4). High constitutive expression of Class IV (along with Class I) in most organs of *A. thaliana* under normal growth conditions has been previously noted [51]. Detailed bioinformatic characterization of genes of LusCTL distinct group should be conducted in future. Probably *LusCTLs* that are highly expressed in fibers may be specific to the gelatinous cell wall, while *LusCTL1* and *LusCTL2* are essential for wall thickening in general.

## Conclusion

High expression of specific *LusCTLs* was observed in different types of thick cell wall producing tissues. *LusCTL1* and *LusCTL2* were preferentially expressed during secondary wall deposition of xylem and were coexpressed with secondary cell wall *CESAs* (*4, 7, 8*). Another group of *LusCTLs*, (especially *LusCTL19, LusCTL20, LusCTL21*) were highly expressed in bast fibers, which have cellulose-rich, gelatinous walls. The group of fiber-enriched LusCTLs was expanded in flax compared to species that do not produce bast fibers, suggesting that these genes might play a unique role during gelatinous cell wall development in general and cellulose synthesis in particular. It is possible that the presence of fiber-specific *LusCTLs*, along with other key participants, determines differences in mechanisms of xylan and gelatinous cell wall formation. To establish the functions of these *LusCTLs* further characterization, including analysis of enzyme activity and structure, is necessary. Chitinase-like proteins remain one the most mysterious proteins in the plant cell wall. This study provides further evidence of their involvement in the process, and distinguishes between groups of CTLs involved in different type of cell wall development.

## Supporting Information

File S1 **LusCTL gene sequences.**
(XLSX)

## References

[1] Gorshkova TA, Gurjanov OP, Mikshina PV, Ibragimova NN, Mokshina NE, et al (2010) Specific type of secondary cell wall formed by plant fibers. Russian Journal of Plant Physiology 57: 328–341.

[2] Mellerowicz EJ, Gorshkova TA (2012) Tensional stress generation in gelatinous fibres: a review and possible mechanism based on cell-wall structure and composition. Journal of Experimental Botany 63: 551–565. 22090441 10.1093/jxb/err339

[3] Andersson-Gunneras S, Mellerowicz EJ, Love J, Segerman B, Ohmiya Y, et al (2006) Biosynthesis of cellulose-enriched tension wood in Populus: global analysis of transcripts and metabolites identifies biochemical and developmental regulators in secondary wall biosynthesis. Plant Journal 45: 144–165. 16367961 10.1111/j.1365-313X.2005.02584.x

[4] Hobson N, Roach MJ, Deyholos MK (2010) Gene expression in tension wood and bast fibres. Russian Journal of Plant Physiology 57: 321–327.

[5] Lafarguette F, Leple JC, Dejardin A, Laurans F, Costa G, et al (2004) Poplar genes encoding fasciclin-like arabinogalactan proteins are highly expressed in tension wood. New Phytologist 164: 107–121. 10.1111/j.1469-8137.2004.01175.x 33873473

[6] Roach MJ, Deyholos MK (2007) Microarray analysis of flax (Linum usitatissimum L.) stems identifies transcripts enriched in fibre-bearing phloem tissues. Molecular Genetics and Genomics 278: 149–165. 17503083 10.1007/s00438-007-0241-1

[7] Mokshina NE, Ibragimova NN, Salnikov VV, Amenitskii SI, Gorshkova TA (2012) Galactosidase of Plant Fibers with Gelatinous Cell Wall: Identification and Localization. Russian Journal of Plant Physiology 59: 246–254.

[8] Roach MJ, Mokshina NY, Badhan A, Snegireva AV, Hobson N, et al (2011) Development of Cellulosic Secondary Walls in Flax Fibers Requires beta-Galactosidase. Plant Physiology 156: 1351–1363. 21596948 10.1104/pp.111.172676 PMC3135967

[9] Wu B, Zhang BC, Dai Y, Zhang L, Shang-Guan KK, et al (2012) Brittle Culm15 Encodes a Membrane-Associated Chitinase-Like Protein Required for Cellulose Biosynthesis in Rice. Plant Physiology 159: 1440–1452. 22665444 10.1104/pp.112.195529 PMC3425189

[10] Zhang DS, Hrmova M, Wan CH, Wu CF, Balzen J, et al (2004) Members of a new group of chitinase-like genes are expressed preferentially in cotton cells with secondary walls. Plant Molecular Biology 54: 353–372. 15284492 10.1023/B:PLAN.0000036369.55253.dd

[11] Kasprzewska A (2003) Plant chitinases - Regulation and function. Cellular & Molecular Biology Letters 8: 809–824. 12949620

[12] Grover A (2012) Plant Chitinases: Genetic Diversity and Physiological Roles. Critical Reviews in Plant Sciences 31: 57–73.

[13] Hermans C, Porco S, Verbruggen N, Bush DR (2010) Chitinase-Like Protein CTL1 Plays a Role in Altering Root System Architecture in Response to Multiple Environmental Conditions. Plant Physiology 152: 904–917. 20007445 10.1104/pp.109.149849 PMC2815904

[14] Sanchez-Rodriguez C, Bauer S, Hematy K, Saxe F, Ibanez AB, et al (2012) CHITINASE-LIKE1/POM-POM1 and Its Homolog CTL2 Are Glucan-Interacting Proteins Important for Cellulose Biosynthesis in Arabidopsis. Plant Cell 24: 589–607. 22327741 10.1105/tpc.111.094672 PMC3315235

[15] Zhong RQ, Kays SJ, Schroeder BP, Ye ZH (2002) Mutation of a chitinase-like gene causes ectopic deposition of lignin, aberrant cell shapes, and overproduction of ethylene. Plant Cell 14: 165–179. 11826306 10.1105/tpc.010278 PMC150558

[16] Collinge DB, Kragh KM, Mikkelsen JD, Nielsen KK, Rasmussen U, et al (1993) PLANT CHITINASES. Plant Journal 3: 31–40. 8401605 10.1046/j.1365-313x.1993.t01-1-00999.x

[17] Jitonnom J, Lee VS, Nimmanpipug P, Rowlands HA, Mulholland AJ (2011) Quantum Mechanics/Molecular Mechanics Modeling of Substrate-Assisted Catalysis in Family 18 Chitinases: Conformational Changes and the Role of Asp142 in Catalysis in ChiB. Biochemistry 50: 4697–4711. 21469745 10.1021/bi101362g

[18] Brameld KA, Goddard WA (1998) The role of enzyme distortion in the single displacement mechanism of family 19 chitinases. Proceedings of the National Academy of Sciences of the United States of America 95: 4276–4281. 9539727 10.1073/pnas.95.8.4276 PMC22479

[19] Jiang C, Huang RF, Song JL, Huang MR, Xu LA (2013) Genomewide analysis of the chitinase gene family in Populus trichocarpa. Journal of Genetics 92: 121–125. 23640415 10.1007/s12041-013-0222-6

[20] PA P, SC dV (2002) Arabidopsis Chitinases: A Genomic Survey. The Arabidopsis Book. 1–25.

[21] Hamel F, Boivin R, Tremblay C, Bellemare G (1997) Structural and evolutionary relationships among chitinases of flowering plants. Journal of Molecular Evolution 44: 614–624. 9169553 10.1007/pl00006184

[22] Tang CM, Chye ML, Ramalingam S, Ouyang SW, Zhao KJ, et al (2004) Functional analyses of the chitin-binding domains and the catalytic domain of Brassica juncea chitinase BjCHI1. Plant Molecular Biology 56: 285–298. 15604744 10.1007/s11103-004-3382-1

[23] Kombrink E, Schroder M, Hahlbrock K (1988) SEVERAL PATHOGENESIS-RELATED PROTEINS IN POTATO ARE 1,3-BETA-GLUCANASES AND CHITINASES. Proceedings of the National Academy of Sciences of the United States of America 85: 782–786. 16578829 10.1073/pnas.85.3.782 PMC279639

[24] Stintzi A, Heitz T, Prasad V, Wiedemannmerdinoglu S, Kauffmann S, et al (1993) PLANT PATHOGENESIS-RELATED PROTEINS AND THEIR ROLE IN DEFENSE AGAINST PATHOGENS. Biochimie 75: 687–706. 8286442 10.1016/0300-9084(93)90100-7

[25] Witmer X, Nonogaki H, Beers EP, Bradford KJ, Welbaum GE (2003) Characterization of chitinase activity and gene expression in muskmelon seeds. Seed Science Research 13: 167–177.

[26] Hematy K, Sado PE, Van Tuinen A, Rochange S, Desnos T, et al (2007) A receptor-like kinase mediates the response of Arabidopsis cells to the inhibition of cellulose synthesis. Current Biology 17: 922–931. 17540573 10.1016/j.cub.2007.05.018

[27] Hong SW, Lee U, Vierling E (2003) Arabidopsis hot mutants define multiple functions required for acclimation to high temperatures. Plant Physiology 132: 757–767. 12805605 10.1104/pp.102.017145 PMC167015

[28] Haigler CH, Zhang DH, Wilkerson CG (2005) Biotechnological improvement of cotton fibre maturity. Physiologia Plantarum 124: 285–294.

[29] Robert S, Mouille G, Hofte H (2004) The mechanism and regulation of cellulose synthesis in primary walls: lessons from cellulose-deficient Arabidopsis mutants. Cellulose 11: 351–364.

[30] Taylor NG, Gardiner JC, Whiteman R, Turner SR (2004) Cellulose synthesis in the Arabidopsis secondary cell wall. Cellulose 11: 329–338.

[31] Wang ZW, Hobson N, Galindo L, Zhu SL, Shi DH, et al (2012) The genome of flax (Linum usitatissimum) assembled de novo from short shotgun sequence reads. Plant Journal 72: 461–473. 22757964 10.1111/j.1365-313X.2012.05093.x

[32] Gorshkova TA, Sal’nikov VV, Chemikosova SB, Ageeva MV, Pavlencheva NV, et al (2003) The snap point: a transition point in Linum usitatissimum bast fiber development. Industrial Crops and Products 18: 213–221.

[33] Kumar M, Thammannagowda S, Bulone V, Chiang V, Han KH, et al (2009) An update on the nomenclature for the cellulose synthase genes in Populus. Trends in Plant Science 14: 248–254. 19375973 10.1016/j.tplants.2009.02.004

[34] S K, K T, M N (1993) MEGA: Molecular Evolutionary Genetics Analysis package, version 1.02. University Park, PA, USA: Pennsylvania State University.

[35] Tamura K, Peterson D, Peterson N, Stecher G, Nei M, et al (2011) MEGA5: Molecular Evolutionary Genetics Analysis Using Maximum Likelihood, Evolutionary Distance, and Maximum Parsimony Methods. Molecular Biology and Evolution 28: 2731–2739. 21546353 10.1093/molbev/msr121 PMC3203626

[36] Huis R, Hawkins S, Neutelings G (2010) Selection of reference genes for quantitative gene expression normalization in flax (*Linum usitatissimum* L.). Bmc Plant Biology 10. 10.1186/1471-2229-10-71 PMC3095345 20403198

[37] Livak KJ, Schmittgen TD (2001) Analysis of relative gene expression data using real-time quantitative PCR and the 2(T)(-Delta Delta C) method. Methods 25: 402–408. 11846609 10.1006/meth.2001.1262

[38] Punta M, Coggill PC, Eberhardt RY, Mistry J, Tate J, et al (2012) The Pfam protein families database. Nucleic Acids Research 40: D290–D301. 22127870 10.1093/nar/gkr1065 PMC3245129

[39] Ageeva MV, Petrovska B, Kieft H, Sal’nikov VV, Snegireva AV, et al (2005) Intrusive growth of flax phloem fibers is of intercalary type. Planta 222: 565–574. 16215710 10.1007/s00425-005-1536-2

[40] Gorshkova T, Morvan C (2006) Secondary cell-wall assembly in flax phloem fibres: role of galactans. Planta 223: 149–158. 16362330 10.1007/s00425-005-0118-7

[41] Somerville C (2006) Cellulose synthesis in higher plants. Annual Review of Cell and Developmental Biology. Palo Alto: Annual Reviews. 53–78. 10.1146/annurev.cellbio.22.022206.160206 16824006

[42] T R (2000) Higher plant cellulose synthases. Genome Biology 1: 1–6. 11178255 10.1186/gb-2000-1-4-reviews3001 PMC138876

[43] Grushetskaya ZE, Lemesh VA, Khotyleva LV (2010) Development of Specific and Degenerate Primers for CesA Genes Encoding Cellulose Synthase in Flax (Linum Usitatissimum L.). Cytology and Genetics 44: 195–199. 20722279

[44] Desprez T, Juraniec M, Crowell EF, Jouy H, Pochylova Z, et al (2007) Organization of cellulose synthase complexes involved in primary cell wall synthesis in Arabidopsis thaliana. Proceedings of the National Academy of Sciences of the United States of America 104: 15572–15577. 17878303 10.1073/pnas.0706569104 PMC2000492

[45] Taylor NG, Howells RM, Huttly AK, Vickers K, Turner SR (2003) Interactions among three distinct CesA proteins essential for cellulose synthesis. Proceedings of the National Academy of Sciences of the United States of America 100: 1450–1455. 12538856 10.1073/pnas.0337628100 PMC298793

[46] Taylor NG, Laurie S, Turner SR (2000) Multiple cellulose synthase catalytic subunits are required for cellulose synthesis in Arabidopsis. Plant Cell 12: 2529–2539. 11148295 10.1105/tpc.12.12.2529 PMC102235

[47] Aspeborg H, Schrader J, Coutinho PM, Stam M, Kallas A, et al (2005) Carbohydrate-active enzymes involved in the secondary cell wall biogenesis in hybrid aspen. Plant Physiology 137: 983–997. 15734915 10.1104/pp.104.055087 PMC1065399

[48] van Hengel AJ, Tadesse Z, Immerzeel P, Schols H, van Kammen A, et al (2001) N-acetylglucosamine and glucosamine-containing arabinogalactan proteins control somatic embryogenesis. Plant Physiology 125: 1880–1890. 11299367 10.1104/pp.125.4.1880 PMC88843

[49] Wojtaszek P, Bolwell GP (1995) SECONDARY CELL-WALL-SPECIFIC GLYCOPROTEIN(S) FROM FRENCH BEAN HYPOCOTYLS. Plant Physiology 108: 1001–1012. 7630932 10.1104/pp.108.3.1001 PMC157450

[50] van Loon LC, Rep M, Pieterse CMJ (2006) Significance of inducible defense-related proteins in infected plants. Annual Review of Phytopathology. 135–162. 10.1146/annurev.phyto.44.070505.143425 16602946

[51] Takenaka Y, Nakano S, Tamoi M, Sakuda S, Fukamizo T (2009) Chitinase Gene Expression in Response to Environmental Stresses in Arabidopsis thaliana: Chitinase Inhibitor Allosamidin Enhances Stress Tolerance. Bioscience Biotechnology and Biochemistry 73: 1066–1071. 10.1271/bbb.80837 19420714

